# Histomorphological Spectrum of Soft Tissue Tumors at a Tertiary Care Hospital

**DOI:** 10.7759/cureus.102552

**Published:** 2026-01-29

**Authors:** Pratibha Meena, Deepshikha Verma, Tina Rai, Shailendra Meena

**Affiliations:** 1 Department of Pathology, Atal Bihari Vajpayee Government Medical College, Vidisha, IND; 2 Department of Preventive Medicine, Atal Bihari Vajpayee Government Medical College, Vidisha, IND

**Keywords:** fnclcc, histopathology, liposarcoma, soft tissue tumor, tumor morphology

## Abstract

Background

Soft tissue tumors (STT) comprise a broad and heterogeneous group of mesenchymal neoplasms with varied histological patterns and biological behavior. Accurate histomorphological evaluation and grading are essential for appropriate diagnosis, prognostication, and management, particularly in malignant cases.

Objectives

The present study was undertaken to analyze the histomorphological spectrum of soft tissue tumors encountered at a tertiary care hospital, to classify them according to the World Health Organization (WHO) 2022 classification, and to grade malignant tumors using the Fédération Nationale des Centres de Lutte Contre le Cancer (FNCLCC) grading system.

Methods

This combined retrospective and prospective descriptive study included 125 cases of soft tissue tumors received in the department of pathology of a tertiary care center over a one-year period. Clinical details were retrieved from requisition forms. Histopathological examination was performed on hematoxylin and eosin-stained sections, and tumors were classified based on WHO criteria. FNCLCC grading was applied to all malignant tumors. Immunohistochemistry (IHC) was performed wherever feasible to support diagnosis.

Results

Out of 125 cases, benign tumors constituted the majority at 107 cases (85.6%), followed by malignant tumors at 15 cases (12.0%) and intermediate tumors at three cases (2.4%). Patients ranged in age from five to 78 years, with a mean age of 39.6 ± 16.8 years. A slight male predominance was observed. Adipocytic tumors were the most common histological subtype, followed by fibroblastic and myofibroblastic tumors. Among malignant tumors, fibrosarcoma was the most frequent subtype. FNCLCC Grade 3 sarcomas were most common, indicating a predominance of high-grade malignancies.

Conclusion

Benign soft tissue tumors were far more common than malignant and intermediate tumors in the present study. Adipocytic tumors formed the largest histological category, while high-grade sarcomas predominated among malignant cases. Detailed histomorphological evaluation, supported by FNCLCC grading, remains crucial for the accurate classification and prognostic assessment of soft tissue tumors.

## Introduction

Soft tissue tumors (STT) are defined as tumors arising from non-epithelial, extraskeletal tissues of the body, excluding the central nervous system and hematolymphoid tissues [1,2]. These include tumors originating from muscular, fibrous, elastic, vascular, and adipose tissues. Tumors arising from peripheral nerves that present as soft tissue masses are also included in this group. Embryologically, soft tissue tumors are primarily of mesodermal origin, with some contribution from neuroectodermal tissue [2].

Soft tissue tumors constitute an immensely diverse group of neoplasms. According to the World Health Organization (WHO) classification of soft tissue tumors of 2022, these tumors are histologically classified based on their resemblance to adult tissue types. They are broadly categorized into benign, intermediate, and malignant tumors within various histological categories. Benign tumors have a limited capacity for autonomous growth, closely resemble the tissue of origin, and show a low tendency for local invasion and recurrence following treatment.

Malignant soft tissue tumors, also known as sarcomas, are aggressive neoplasms with the ability to invade surrounding tissues and a propensity for recurrence and metastasis. Radical surgical excision is often required; however, it does not guarantee the complete prevention of recurrence. Certain tumors fall into an intermediate or borderline category, in which the assessment of malignant potential may be challenging. The annual worldwide incidence of soft tissue tumors is approximately six per 100,000 population [2,3]. Benign soft tissue tumors are more common than malignant ones, with a reported ratio of 100:11 [4].

The etiology of soft tissue tumors remains largely obscure, though risk factors such as viral infections, radiation exposure, chemical carcinogens, inherited syndromes, and genetic mutations have been implicated in their pathogenesis [5]. Histological diagnosis based on the light microscopic examination of hematoxylin and eosin-stained sections, along with special stains, forms the basis for the application of ancillary techniques such as immunohistochemistry (IHC), electron microscopy, cytogenetics, and flow cytometry.

This study aims to evaluate the histomorphological spectrum of soft tissue tumors reported at a tertiary care hospital catering to a significant population of central Madhya Pradesh, to grade sarcomas according to the Fédération Nationale des Centres de Lutte Contre le Cancer (FNCLCC) grading system, and to analyze immunohistochemical features wherever feasible.

## Materials and methods

A retrospective and prospective study was done on patients whose specimens were sent for histopathological examination to the department of pathology of Atal Bihari Vajpayee Government Medical College, Vidisha, over the period of one year, that is, from October 2024 to October 2025. A total of 125 cases were diagnosed as soft tissue tumors. These reported cases were analyzed in correlation with the information of age, sex, size, and location retrieved from the requisition forms of the patients. The microscopic findings of the histological sections of these cases were studied in detail in hematoxylin and eosin stain. The histological subtyping of these tumors was done according to the WHO classification of soft tissue tumors of 2022. All the sarcomas were categorized according to the FNCLCC reporting system, as shown in Table 1 [6]. The Institutional Ethical Committee of Atal Bihari Vajpayee Government Medical College, Vidisha, issued approval 10/B/7/25/IEC/ABVGMC/VIDISHA/2025.

**Table 1:** FNCLCC reporting system for sarcomas Source: [6] FNCLCC, Fédération Nationale des Centres de Lutte Contre Le Cancer; HPF, high-power field

Tumor Differentiation	
Score 1	Sarcoma closely resembling normal adult mesenchymal tissue (e.g., well-differentiated liposarcoma)
Score 2	Sarcomas for which histological typing is certain (e.g., myxoid liposarcoma)
Score 3	Embryonal and undifferentiated sarcomas, sarcomas of doubtful type, and synovial sarcomas
Mitotic Count	
Score 1	0-9 mitoses per 10 HPF
Score 2	10-19 mitoses per 10 HPF
Score 3	≥20 mitoses per 10 HPF
Tumor Necrosis	
Score 1	No necrosis
Score 2	<50% tumor necrosis
Score 3	≥50% tumor necrosis

The gross findings and microscopic pathological findings were evaluated in detail on hematoxylin and eosin-stained slides. Histological subtyping was done according to the WHO classification of soft tissue tumors (STT) of 2022. The IHC study will be done wherever possible in malignant cases reported for correlation.

All the cases of soft tissue tumors whose samples were sent for histopathological examination to our department were included in the present study, while patients with a prior history of chemotherapy or radiotherapy for any soft tissue tumor, all non-mesenchymal tumors, and bone tumors were excluded from the study.

Sample size calculation

As this was a hospital-based descriptive study with a fixed duration of one year, a formal sample size calculation was not performed. All consecutive cases of soft tissue tumors received for histopathological examination in the department of pathology during the study period were included. Thus, the final sample size comprised 125 cases.

Statistical analysis

The data were compiled and entered into a spreadsheet (Microsoft Excel, Microsoft Corp., Redmond, WA) and then exported to the data editor of IBM SPSS Statistics for Windows, version 20 (released 2011; IBM Corp., Armonk, NY). Quantitative variables were summarized as means and standard deviations.

## Results

This study was done by analyzing the histopathological sections of cases, reported as soft tissue tumors, in the department of pathology of a tertiary care hospital. A total of 125 cases were included in the study. The majority of the soft tissue tumors were benign (107 cases, 85.6%), followed by malignant (15 cases, 12%) and intermediate tumors (three cases, 2.4%) (Table 2).

**Table 2:** Nature of the tumor

Category	Frequency	Percentage
Benign	107	85.6%
Intermediate	3	2.4%
Malignant	15	12%
Total	125	100%

The age of the patients ranged from five to 78 years, with a mean age of 39.6 ± 16.8 years. Men constituted 54.4% of the cases, while women accounted for 45.6% (Table 3).

**Table 3:** Demographic details Age is expressed as mean ± standard deviation. Gender is expressed as frequency and percentage

Variables	Value
Age range (in years)	5-78
Mean age (in years)	39.6 ± 16.8
Gender: male	68 (54.4%)
Gender: female	57 (45.6%)

Adipocytic tumors constituted the most common histological category (45 cases, 36.0%), followed by fibroblastic and myofibroblastic tumors (28 cases, 22.4%). Vascular tumors accounted for 12.0% of cases (15 cases), while tumors of uncertain differentiation were the least common (six cases, 4.8%) (Table 4).

**Table 4:** Histological types of tumor

Serial number	Histological type	Frequency	Percentage
1	Adipocytic	45	36%
2	Fibroblastic and myofibroblastic	28	22.4%
3	Fibrohistiocytic	12	9.6%
4	Muscular	10	8%
5	Vascular	15	12%
6	Peripheral nerve sheath	9	7.2%
7	Tumors of uncertain differentiation	6	4.8%
	Total	125	100%

In the female population, the most common type of soft tissue tumor reported was smooth muscle tumors, followed by adipocytic tumors, whereas in men, adipocytic tumors constituted the majority among all the cases. Among the 15 malignant soft tissue tumors, FNCLCC Grade 3 tumors were the most frequent (seven cases, 46.7%), followed by Grade 2 (six cases, 40.0%) and Grade 1 tumors (two cases, 13.3%). Liposarcoma accounted for all Grade 1 tumors, while undifferentiated pleomorphic sarcoma showed exclusively high-grade morphology (Table 5).

**Table 5:** FNCLCC grading In malignant soft tissue tumors FNCLCC: Fédération Nationale des Centres de Lutte Contre le Cancer

	FNCLCC Grade 1	FNCLCC Grade 2	FNCLCC Grade 3	Total
Liposarcoma	2	1	0	3
Fibrosarcoma	0	3	2	5
Synovial sarcoma	0	1	1	2
Undifferentiated pleomorphic sarcoma	0	0	2	2
Malignant mesenchymal neoplasm	0	1	2	3
Total	2	6	7	15

## Discussion

A total of 125 cases of soft tissue tumors were analyzed in the present study. Benign tumors constituted the majority of cases, followed by malignant tumors, while intermediate tumors formed the smallest group. In our study, benign soft tissue tumors accounted for 85.6% of the cases (107 cases), malignant tumors for 12.0% (15 cases), and intermediate tumors for 2.4% (three cases). This predominance of benign tumors is consistent with the findings of Tapadar et al. (2021), who reported a higher prevalence of benign soft tissue tumors (89.4%) [7].

In the present study, soft tissue tumors were observed across a wide age range, with a higher frequency noted in young and middle-aged adults. Similar age predilection has been reported by Tapadar et al. (2021), who observed the highest number of cases in the 21-30-year age group [7]. In contrast, Choudhary et al. (2020) reported a peak incidence in the 31-40-year age group [8]. These variations may be attributed to differences in study population, geographic distribution, and referral patterns.

With respect to histological categorization, adipocytic tumors (36.0%, 45 cases) were the most common soft tissue tumors in the present study, followed by fibroblastic and myofibroblastic tumors (22.4%, 28 cases). This finding is in agreement with studies conducted by Pagaro et al., who also identified adipocytic tumors as the most common soft tissue tumors [9]. However, while some studies have reported vascular tumors as the second most common benign soft tissue tumor, fibroblastic and myofibroblastic tumors constituted the second largest group in our study [7,8,10]. These differences may reflect regional variation and institutional referral bias.

Intermediate soft tissue tumors were relatively uncommon in our study, accounting for only three cases (2.4%). These included cases such as dermatofibrosarcoma protuberans, inflammatory myofibroblastic tumor, and solitary fibrous tumor. The low frequency of intermediate tumors has also been reported in other Indian studies and may be due to their relatively rare occurrence compared to benign and malignant counterparts.

Among malignant soft tissue tumors, fibrosarcoma constituted the most common subtype, followed by liposarcoma, synovial sarcoma, undifferentiated pleomorphic sarcoma, and malignant mesenchymal neoplasm. Undifferentiated pleomorphic sarcoma showed a predominantly high-grade morphology, which is in concordance with the findings of Umarani et al. (2015) [11]. A subset of malignant tumors could not be categorized into a specific histological entity and were classified under malignant mesenchymal neoplasm, a finding also reported by other authors [12]. Liposarcomas formed a significant proportion of malignant tumors in the present study, similar to observations made by Umarani et al. (2015) [11].

The majority of the soft tissue tumors were located in the extremities (70%, 88 cases), especially the lower extremities (55%, 69 cases), followed by the trunk and abdomen (25%, 31 cases). Similar findings were reported by Samartha et al. [13], Jain et al. [14], and Chakrabarti et al. [15].

Overall, the findings of the present study highlight the predominance of benign soft tissue tumors, the relative rarity of intermediate tumors, and the predominance of high-grade sarcomas among malignant cases, emphasizing the importance of accurate histomorphological evaluation and grading for appropriate diagnosis and management.

Limitations

The study was conducted at a single tertiary care center with a relatively short study duration. Immunohistochemistry could not be performed in all cases due to limited availability, which may have affected precise subtyping in a few cases.

## Conclusions

Soft tissue tumors represent a highly diverse group of neoplasms that present both diagnostic and therapeutic challenges. A good understanding of their morphology, histology, classification, and grading is important to diagnose and correctly classify these tumors. Evaluating morphological prognostic factors, particularly histological grade, is crucial for the FNCLCC grading system, which is significant in predicting the prognosis of soft tissue sarcomas.
